# Metabolic fingerprinting of dogs with idiopathic epilepsy receiving a ketogenic medium-chain triglyceride (MCT) oil

**DOI:** 10.3389/fvets.2022.935430

**Published:** 2022-10-06

**Authors:** Benjamin Andreas Berk, Claudia Ottka, Tsz Hong Law, Rowena Mary Anne Packer, Annette Wessmann, Andrea Bathen-Nöthen, Tarja Susanna Jokinen, Anna Knebel, Andrea Tipold, Hannes Lohi, Holger Andreas Volk

**Affiliations:** ^1^Department of Clinical Science and Services, Royal Veterinary College, Hatfield, United Kingdom; ^2^BrainCheck.Pet^®^, Tierärztliche Praxis für Epilepsie, Mannheim, Germany; ^3^Department of Veterinary Biosciences and Department of Medical and Clinical Genetics, Folkhälsan Research Center, University of Helsinki, Helsinki, Finland; ^4^PetBiomics Ltd., Helsinki, Finland; ^5^Pride Veterinary Centre, Neurology/Neurosurgery Service, Derby, United Kingdom; ^6^Tierarztpraxis, Dr. A. Bathen-Nöthen, Cologne, Germany; ^7^Department of Equine and Small Animal Medicine, Faculty of Veterinary Medicine, Helsinki, Finland; ^8^Department of Small Animal Medicine and Surgery, University of Veterinary Medicine, Hannover, Germany

**Keywords:** metabolome, neurotransmitter, epilepsy, biomarker, medium-chain triglyceride, canine

## Abstract

Consumption of medium-chain triglycerides (MCT) has been shown to improve seizure control, reduce behavioural comorbidities and improve cognitive function in epileptic dogs. However, the exact metabolic pathways affected by dietary MCT remain poorly understood. In this study, we aimed to identify changes in the metabolome and neurotransmitters levels relevant to epilepsy and behavioural comorbidities associated with the consuming of an MCT supplement (MCT-DS) in dogs with idiopathic epilepsy (IE). Metabolic alterations induced by a commercial MCT-DS in a population of 28 dogs with IE were evaluated in a 6-month multi-centre, prospective, randomised, double-blinded, controlled cross-over trial design. A metabolic energy requirement-based amount of 9% MCT or control oil was supplemented to the dogs' stable base diet for 3 months, followed by the alternative oil for another 3 months. A validated, quantitative nuclear magnetic resonance (NMR) spectroscopy platform was applied to pre- and postprandially collected serum samples to compare the metabolic profile between both DS and baseline. Furthermore, alterations in urinary neurotransmitter levels were explored. Five dogs (30%) had an overall reduction in seizure frequency of ≥50%, and were classified as MCT-responders, while 23 dogs showed a ≤50% reduction, and were defined as MCT non-responders. Amino-acid metabolism was significantly influenced by MCT consumption compared to the control oil. While the serum concentrations of total fatty acids appeared similar during both supplements, the relative concentrations of individual fatty acids differed. During MCT supplementation, the concentrations of polyunsaturated fatty acids and arachidonic acid were significantly higher than under the control oil. β-Hydroxybutyric acid levels were significantly higher under MCT supplementation. In total, four out of nine neurotransmitters were significantly altered: a significantly increased γ-aminobutyric acid (GABA) concentration was detected during the MCT-phase accompanied by a significant shift of the GABA-glutamate balance. MCT-Responders had significantly lowered urinary concentrations of histamine, glutamate, and serotonin under MCT consumption. In conclusion, these novel data highlight metabolic changes in lipid, amino-acid and ketone metabolism due to MCT supplementation. Understanding the metabolic response to MCT provides new avenues to develop better nutritional management with improved anti-seizure and neuroprotective effects for dogs with epilepsy, and other behavioural disorders.

## Introduction

Epilepsy is the most common neurological disorder in dogs affecting an estimated 0.6–0.8% (1–3) of the population in first opinion practise. Epilepsy is defined by its recurrent seizure activity (4), but is often associated with behavioural and cognitive comorbidities (5), such as anxiety (6–10), ADHD-like behaviour (11), deficits in general trainability (8, 12), spatial memory (13, 14) or earlier signs of canine cognitive dysfunction (15). Although there is active international research in this field, therapeutic approaches to canine epilepsy focus on drugs for seizure suppression (16, 17), instead of treating epileptogenesis or the disease itself, including the comorbidities mentioned above (18). The cellular, pathophysiological mechanisms and the biological manifestation of seizures leading to epilepsy are still unclear. However, chronic administration of anti-seizure drugs (ASD), often in tandem as polytherapy, comes with a delicate balance between the desired seizure-suppressive effects and drug-related adverse effects (19). Despite appropriately managed polypharmacotherapy, around one-third of dogs with idiopathic epilepsy continue to have difficulty controlling seizures (20–23). Seizure activity and drug related adverse effects contribute to the reduction in quality of life (QoL) for both dogs and their owners (5, 24–28) emphasising the need for new treatment strategies.

Dietary interventions, such as ketogenic diets (KD) have been explored as a therapeutic option to prevent and treat seizures in humans with epilepsy since the early 1920s. Originally, such diets were designed to mimic the metabolic state with its biochemical changes of fasting, as this has been shown to evoke anticonvulsant properties (29). Since then, dietary modification in seizure control has been extensively studied in human medicine (30–32) and some rodent epilepsy models (33–37), showing varying levels of anti-seizure effects.

In veterinary medicine, dietary manipulations have attracted increasing consideration as an alternative approach to managing seizure activity and behaviour in dogs with idiopathic epilepsy (IE) (11, 38, 39). In 2015, Law et al. compared an MCT kibble diet (test formula contained 5.5 % MCT; 10% of the total formula calories from C8, C10, C12) to a standardised placebo diet on its seizure-controlling effects in 21 chronically non-responsive ASD-treated dogs with IE in a crossover trial design (39). Anti-seizure properties and a significant rise in blood β-hydroxybutyrate (BHB) concentrations were found. In 2020, another study showed that the supplementation of a commercial MCT-oil as 9% of the metabolic energy (ME) rate on a various base diet had proven similar positive anti-seizure effects and resulted in an increase in BHB serum concentrations (40). The in average daily feed ration consisted of at least 50% carbohydrates in both studies. This supports the hypothesis, that medium-chain fatty acids (MCFA), such as caprylic, capric, and lauric fatty acids, as the main constituent within the MCT, may possess direct mechanistic effects in the anticonvulsive properties and come with specific metabolic adaptions (41–46).

Recently, we have shown that a 9% ME MCT supplemented diet has also a positively affects anxious behaviour and cognitive abilities in some dogs with IE (40, 47). However, the knowledge about how MCT alters the metabolism and may lead to these brain effects in dogs is limited. In the past, MCT containing diets has been shown to confer neuroprotective effects in humans (48). The same suggestion has been made in dogs after 3 months of MCT consumption, which led to significant global changes in the lipid metabolism. A considerable increase of the saturated C17:0 fatty acyl moieties (lysophosphatidylcholine, phosphatidylcholine) was highlighted and discussed as associated with the neuroprotective, downstream metabolites triheptanoin (49). Triheptanoin is a triacylglyceride (TAG) consisting of three heptanoate (C7:0) fatty acids (FA). Especially, C17:0 fatty acyl moieties can be metabolised to C7:0 moieties after five cycles of β-oxidation. If it is hypothesised that triheptanoin may influence anaplerotic mechanisms in the brain by delivering tricarboxylic acid cycle substrates and supporting mitochondrial metabolic pathways (37, 50, 51).

Overall, most of the beneficial effects of MCFA have been attributed to ketone body formation. More recent results has raised the question, whether MCFA *per se* and its derived downstream metabolites may play a much more relevant role in the treatment of epilepsy.

This study aimed to explore the metabolomic effects behind an oral MCT oil supplementation in epileptic dogs governing, as previously reported, anti-epileptic and pro-cognitive properties. Thus, appropriate physicochemical methods were chosen to identify, and measure altered metabolites in serum and urine putting them into a physiological context.

## Materials and methods

This clinical study was conducted following the guidelines of the International Cooperation of Harmonization of Technical Requirements for Registration of Veterinary Products (VICH) GL9 Good Clinical Practices (GCP) and the European Agency for the evaluation of Medical Products (EMEA). This study was approved by the Royal Veterinary College Clinical Research Ethical Review Board (CRERB) (URN 2016 1558).

### The clinical trial

**Figure 1:**
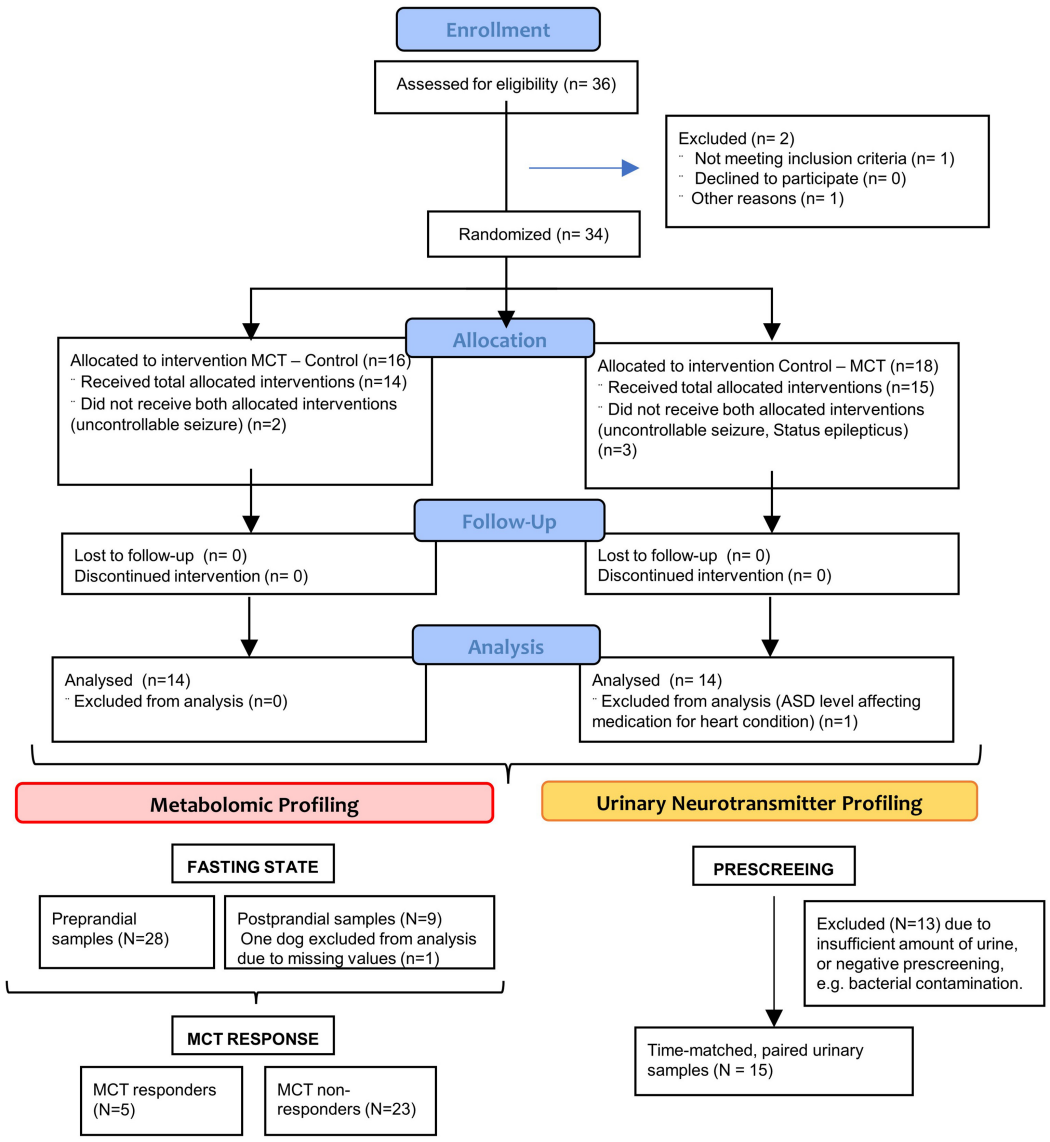
CONSORT flow diagram—Flow diagram of the progress through the phases of this randomized clinical trial of two groups (that is, as shown, enrolment, intervention allocation, follow-up, and data analysis).

A 6-month multicenter, prospective, randomised, double-blinded, controlled, dietary crossover trial was conducted to investigate the short-term effects of an MCT oil supplement to a “control” dietary oil supplement (DS) for the management of canine epilepsy and their correlation with metabolic responses caused by this dietary modification (Figure 1). The clinical study design has been published separately in detail (52), as were the results of the effect of the supplement on seizure frequency (40) and cognition (47). Briefly, dogs diagnosed with IE (International Veterinary Epilepsy Task Force tier II level diagnosis) and not responding to at least one appropriate chosen and administered ASD were recruited at five different study centres in three countries for this dietary trial. Each dog was randomly assigned a first DS-1, either the MCT- or control-DS, instructed to supplement alongside their normal diet and fed initially for 3 months (day 1 to day 90 ± 2), followed by a respective switch to the second DS-2 for another 3 months (day 90 to day 187 ± 2). The first 7 days of dietary crossover, DS period 2 (day 90 to day 97 ± 2) were considered as wash-out period and allowed metabolic adaption.

### Dietary supplementation and test products

A total of 9% of the daily metabolic energy requirement was translated into the individual oil volume prescribed per day and added on the top of their usual energy amount. Throughout the study, all dogs were fed twice daily with their regular diet receiving half amount of the total daily oil requirement. ASD treatment was not withheld at any point nor changed in this study. Concomitant changes in ASD medication or diet during participation led to study exclusion.

Both used oils were commercially available dietary supplements suitable for human consumption. While the test oil was an MCT-DS purified from palm and rapeseed oil containing high levels of two medium-chain fatty acids (MCFA) 50–65% caprylic acid (C8) and 30–50% capric acid (C10) (End GmbH, Germany, Batch No. L 16 M12), the control oil was colourless, extra-virgin olive oil with 8 kcal per ml containing 11% saturated, 11% polyunsaturated, and 78% g monounsaturated fat (Filippo Berio, Italy, Batch No LE194M04). To ensure a throughout blinded study design, both oils were dispensed in brown bottles and all owners were instructed on how to store and supplement. The base diet was recorded, but not individually assessed.

### Sample collection and preparation

Serum and urine samples were collected based on a standard operating procedure for different metabolomic approaches to interrogate the changes in metabolic profile seen in epileptic dogs receiving a daily ketogenic DS onto a stable base diet and anticonvulsive medication regime (Figure 1).

#### Urine samples

On the morning of each scheduled study visit (V1–V3), time-matched urine samples were collected from each dog using the free catch method, and transferred into sterile urine tubes. The samples were identified as baseline, control DS phase or MCT-DS phase samples. As quality pre-screening standard, part of the urine was subsequently analysed for urine specific gravity, pH, nitrite, protein, ketones, bilirubin, bacteria, crystals, and blood (erythrocytes, leukocytes) at two local laboratories (Diagnostic Laboratory Services, Royal Veterinary College, United Kingdom; IDEXX Laboratories, Inc., Ludwigsburg, Germany). Only dogs with unremarkable urine analysis at all study visits were included into further analysis. Aliquots with 500 ul of urine were prepared and stored in cryovials at −80°C up to trial completion.

#### Blood samples

At each study visit (V1–V3), two blood samples were collected from each dog. A pre-prandial sample was taken after 12 h of overnight fasting and the second in the post-prandial state 2 h after feeding the dog with its usual diet supplemented with the prescribed volume of DS. Routine anti-seizure medication was also given during feeding. Both samples were taken into both clotting activator dipotassium EDTA-containing (with serum-separation gel) polypropylene blood-collection tubes and plain polypropylene blood-collection tubes (International Scientific Supplies Ltd). Serum tubes were left to coagulate on ice for 15 min prior to centrifugation. The samples were centrifuged for 10 min at 2,000 × g at 2–5°C, and the resulting supernatant (=serum) was separated. Serum was stored in cryovials at −80°C up to study completion. Only dogs with a total amount of 300 ul per cryovial for each study visit were finally studied using a targeted metabolomics approach.

### Metabolomic exploration methods

Two analytical methods [nuclear magnetic resonance (NMR) and high-performance liquid chromatography (HPLC)] were used to acquire metabolic data in serum and urine (66).

#### Neurotransmitter profiles in urine

Urine samples (~2 mL) were sent to Doctors Data Inc. (St. Charles, IL) where they were stored at −80°C until analysis. The urine specimens were diluted according to their creatinine level. The diluted urine samples were mixed with internal standards and derivatized to ethoxycarbonyl-ethyl-ester derivatives with ethyl-chloroformate and ethanol-pyridine at room temperature. The derivatives of these neuro-biogenic amines were then extracted by polymeric reversed phase SPE cartridge to remove protein and water-soluble impurities. The analytes were analysed by Agilent 6460 liquid chromatography–mass spectrometry (LC-MS/MS) with Jet Stream (AJS) Electrospray Ionisation. Multiple reaction monitoring (MRM) transition ions were detected by MS/MS technique for each analyte of interest while a precursor ion is further broken down to a fragment ion for greatly improved selectivity. The chromatography separation was achieved on a Poroshell 120 EC-C8, 2.1 × 150 mm, 2.7 μM (Agilent Technologies, Palo Alto, CA). The urine-based concentration of the following neurotransmitters relevant for epilepsy and behavioural comorbidities were then quantified for statistical assessment: serotonine, dopamine, epinephrine, norephinephrine, glutamate, GABA, histamine, glycine, phenethylamine.

#### Metabolic profiles in serum

Both the pre- and post-prandial samples were measured on each visit day. While pre-prandial samples were used to elucidate the global shifts in metabolism, post-prandial samples were used for elucidating the immediate and short-term metabolic responses associated with DS consumption. Sample analysis was conducted using a validated, canine-specific proton nuclear magnetic resonance (^1^H NMR) spectroscopy-based metabolomics platform quantifying 123 measurands (53). Sample preparation with buffer allocation was performed by a PerkinElmer 8-tip JANUS automated liquid handler (Perkin-Elmer Inc., Waltham, Massachusetts, United States) and nuclear magnetic resonance (NMR) measurement utilising a Bruker AVANCE III HD 500 spectrometer with a 5 mm triple-channel (1H, 13C, 15N) z-gradient Prodigy probe head (Bruker Biospin, Rheinstetten, Germany) with principles outlined in Soininen and colleagues (53, 54).

#### Ketone body measurements in serum

Pre- and post-prandial β-Hydroxybutyric acid (BHB) concentrations were measured on each visit day from the collected pre- and post-prandial serum samples. Th serum samples were shipped for subsequent analysis for BHB concentrations at one of the local laboratories (Diagnostic Laboratory Services, Royal Veterinary College, United Kingdom; IDEXX Laboratories, Inc., Ludwigsburg, Germany).

### Statistical analysis

For all above-mentioned neurotransmitters in urine and metabolites in serum, (a) concentrations, (b) the percentage change relative to baseline and (c) specific ratios were statistically compared. Comparisons were made between the following two subgroups: (i) baseline and/ or type of DS; (ii) fasting state: pre- vs. post-prandial state; (iii) MCT response rate, based on reduction in seizure frequency defined as the number of seizures per month [subclassified as “R” (R) ≥ 50% reduction, “NR” (NR) ≤50% reduction], as reported previously (40).

Baseline and DS concentrations were compared using a two-sided, match-paired Student's *t*-test for parametric data and Wilcoxon matched-pairs signed-rank test for non-parametric data. When comparing MCT R with NR, unpaired two-sided *t*-tests for parametric data and Mann-Whitney U-test for non-parametric data were chosen (GraphPad Prism^®^ STATCON GmbH; Schulstr. 2; 37213 Witzenhausen, Germany). To account for multiple comparisons. *P*-value correction was conducted using false discovery rate (FDR) (55). The relationships between selected pairs of continuous variables were analysed using Pearson's correlation coefficient analysis. Non-parametric data are presented as median (25th−75th percentile), and parametric data are represented as mean values and standard deviations.

## Results

### Animal population

As formerly reported (40), the clinical trial was completed by 28 of the 36 recruited dogs, which included 18 different breeds at five different study sides in three different countries [UK (*N* = 16), Germany (*N* = 14); Finland (*N* = 1)]. The study population consisted of 16 males, of which were 12 neutered and 4 were intact, and 12 females, of which 10 were neutered and 2 intact. The population of dogs were on average 5.46 ± 2.61 years of age and weighed 25.6 ± 13.4 kilogrammes at the start of the trial. Twenty-five (89%) of the 28 dogs received phenobarbital (PB) as ASD, while 32% received a second and 61% (*N* = 17) a third ASD as combination therapy. Predominantly, potassium bromide or levetiracetam was given as add-on medication (40%). During MCT supplementation a reduction in seizure frequency was observed in over 60% of dogs (*N* = 17). However, only thirty percent of dogs (*N* = 5) had an overall reduction in seizure frequency ≥50% and were from there classified as MCT responders (R). Thus, 23 dogs were grouped to MCT non-responders (NR). Twelve dogs showed ≤50% (21%; 6–42%) reduction, while three dogs had no change and eight dogs showed an overall increase in seizure frequency per month (8%; 2–33%), as reported previously (40).

Urine samples of 15 dogs (53%) were available in sufficient quantity and were sent for neurotransmitter level quantification. This subpopulation consisted dominantly of male-neutered (40%), pure-breeds (80%) receiving PB in 87% as ASD. The mean age was 5.75 ± 2.14 years of age and the mean weight was 26.1 ± 15.12 kilogrammes at the start of the trial. All five MCT-R were included (30%, *N* = 5), while the rest were classified as NR (70%, *N* = 10).

### Feed ration

Most dogs (82%, *N* = 19) received a commercial dry food as a single diet (46%) or in combination with wet food (11%), raw feed (14%) or home-cooked add-on (11%). A variable self-cooked food or raw feed ration was given to the rest (*N* = 8). Fourteen out of 28 dogs were supplemented first with the MCT-DS, while 15 got the control-DS first, subsequently followed by the dietary switch to the other DS type.

### Urinary neurotransmitter profile

**Figure 2:**
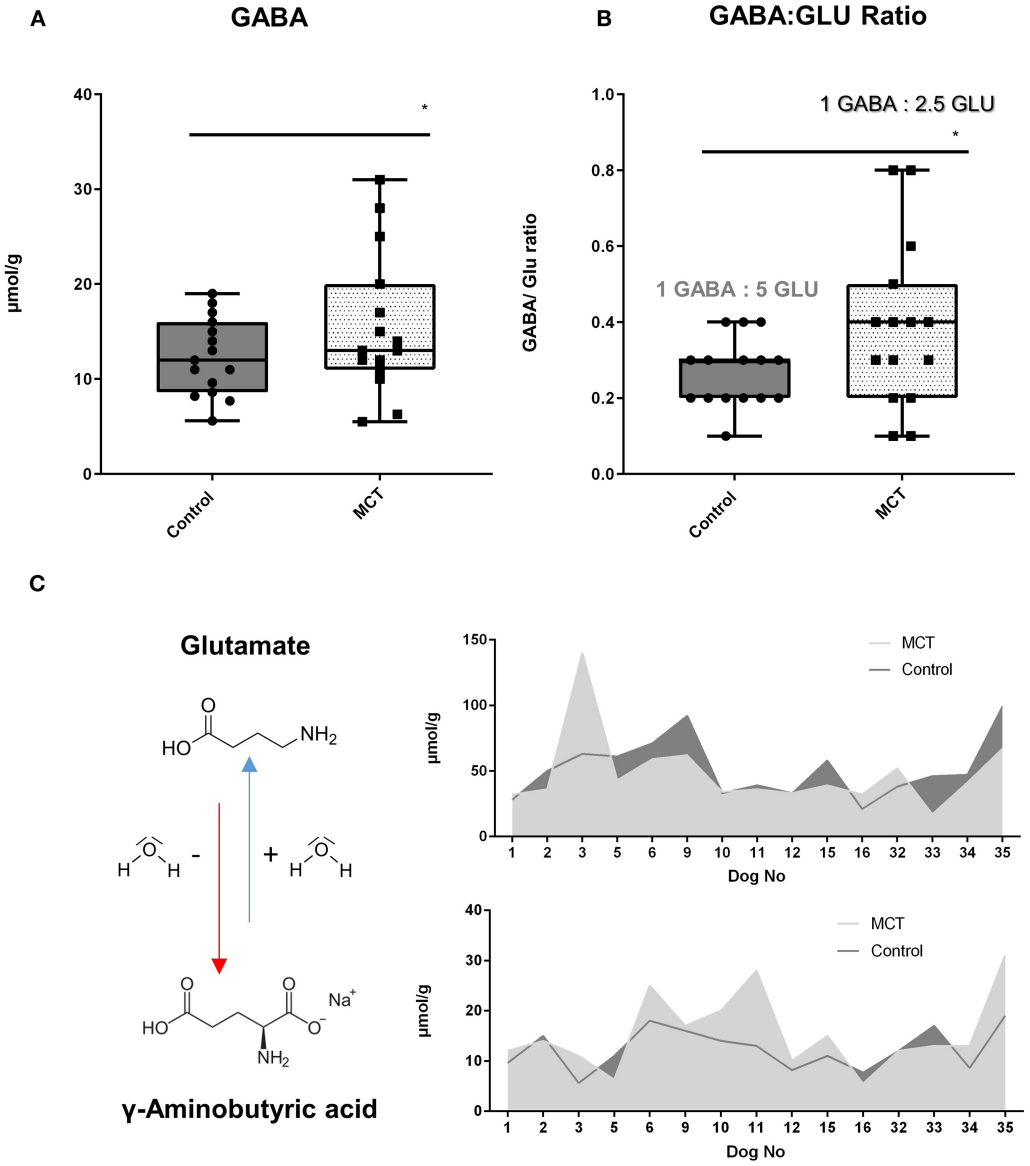
Urinary neurotransmitter profile: Effect of the medium-chain TAG dietary supplement (MCT-DS, light pointed) on the γ-Aminobutyric acid (GABA)—glutamate balance in urine compared with the control dietary supplement (Control-DS, dark-grey). Significant **(A)** increased γ-aminobutyric acid (GABA) concentrations (*P* = 0.0044) and a shift in the GABA/glutamate ratio **(B)** during the MCT phase was found (*P* = 0.0025). A predominant shift to the GABAeric side in the GABA/glutamate ratio **(C)** was observed under MCT-DS consumption (light grey) compared to the Control DS (dark-grey). Data are shown as box-and-whisker plots (central lines of the box represent the median, lower and upper limits of the box represent the 25th and 75th percentiles and whiskers represent the minimum and maximum). Two-sided Wilcoxon's matched-pairs rank tests were used to compare control and MCT-DS groups. *P < 0.05.

Significant differences in four out of nine neurotransmitters were observed in dogs consuming the control-DS and MCT-DS after 90 days of DS consumption (Supplementary Table 1). In all 15 dogs, a significant increased γ-aminobutyric acid (GABA) concentration (*p* = 0.044) was detected during the MCT-phase (Figure 2A). This increase was accompanied by a significant shift in the balance between GABA and glutamate (Figure 2B). Supplementation of MCT has resulted in a relative increase of 50% on the GABAergic side (*p* = 0.025) (Figures 2B,C). Moreover, significant differences were observed between R and NR during the MCT-DS phase. MCT-R were also found to have significantly lowered urinary concentrations of histamine (*p* = 0.006), glutamate (*p* = 0.046) and serotonin (*p* = 0.012) during the MCT-DS phase, but not during the control-DS phase [histamine (*p* = 0.345); glutamate (*p* = 0.323); serotonin (*p* = 0.269)]. The same was found for all three metabolites when compared to baseline.

### Serum metabolite concentrations

Similarly, to the urinary neurotransmitters, significant differences in metabolite profiles were observed both between the MCT-DS and control-DS supplemented dogs after 90 days of supplementation, and between R and NR during dietary supplementation with MCT-DS (Supplementary Tables 2–4, in SI units).

#### Consumption of MCT-DS

**Figure 3:**
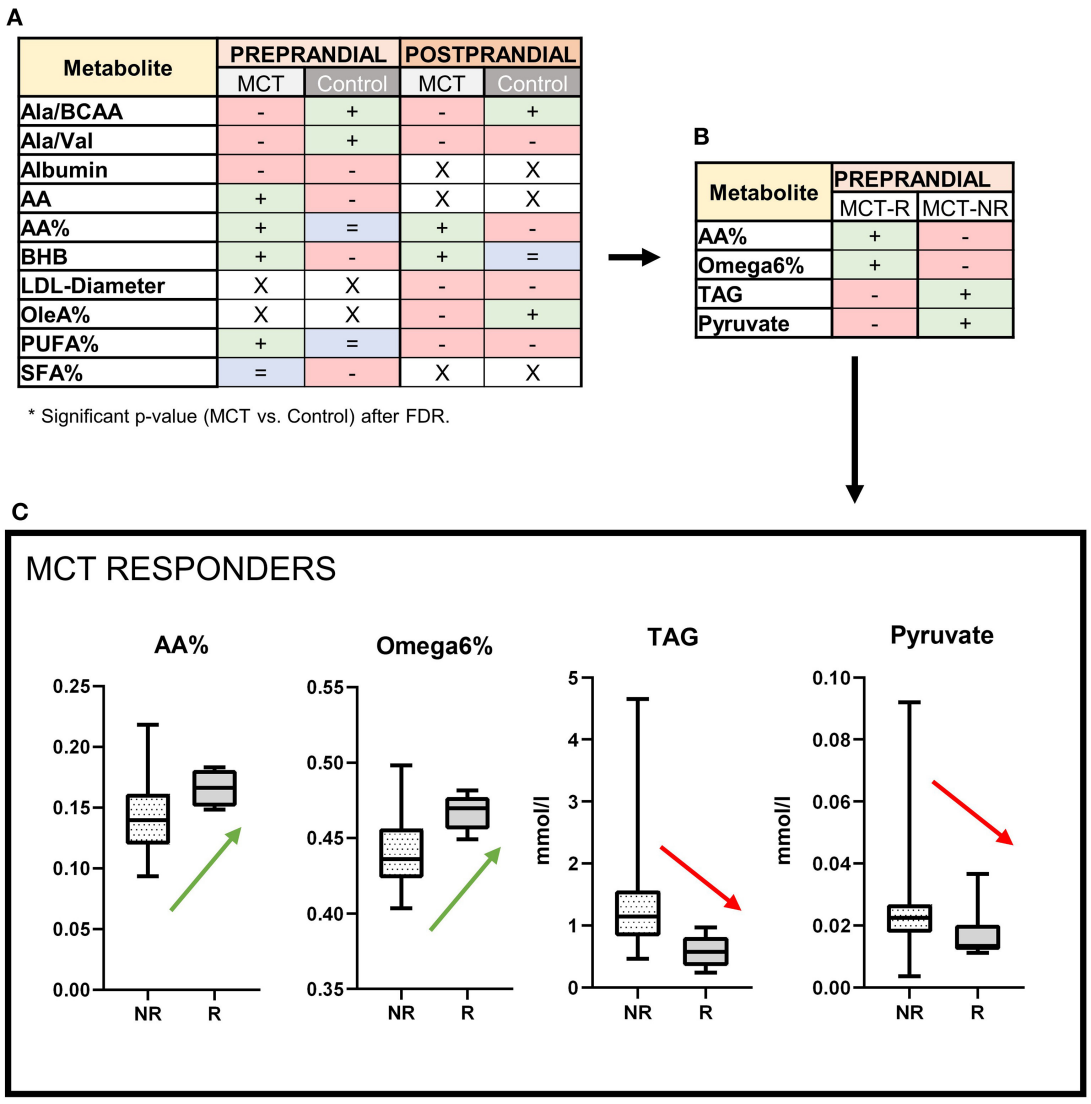
Serum metabolite profile: Effects of the medium-chain TAG dietary supplement (MCT-DS, light-grey) on serum metabolites compared with the control dietary supplement (Control-DS, dark-grey) in **(A)** pre-prandial (light-orange) and post-prandial (dark-orange) state (*N* = 28). If significant between both dietary supplements **(A)**, altered parameters are labelled according to their change related to baseline (red = decreased, blue = stable, green = increased, x = not significant). MCT-R **(B)** showed significant abundances in four different metabolites. MCT-R **(C)** show a shift in their fatty acid profiles to increased serum levels of arachidonic acid (*P* = 0.0283) and Ω 6 fatty acids (*P* = 0.0171), but decreased levels of triglycerides (*P* = 0.0034) and pyruvate (*P* = 0.0386). Data are shown as box-and-whisker plots (central lines of the box represent the median, lower and upper limits of the box represent the 25th and 75th percentiles and whiskers represent the minimum and maximum). Two-sided Wilcoxon's matched-pairs rank tests were used to compare control and MCT-DS groups. *P < 0.05.

Changes in lipid, amino-acid and ketone metabolism were observed during MCT consumption, as several metabolite concentrations and ratios were significantly altered (Figure 3A).

In pre-prandial state, seven metabolites and six ratios were altered with statistical significance between Control DS and MCT DS phase (Supplementary Table 2). While two amino acid ratios were shown to be decreased related to baseline [ratio of alanine to total branched-chain amino acids (Ala/BCAA), ratio of alanine to valine (Ala/Val)], a significantly increased concentration of Arachidonic acid (AA) (*p* = 0.0464) was found. Concerning to the total fatty acids amount, proportional (%) concentrations of AA (*p* = 0.0081) and PUFA (*p* = 0.0162) were significantly elevated, while saturated fatty acids (SFA, *p* = 0.0450) were lowered in comparison to the Control DS. BHB serum concentrations were significantly increased by MCT oil (*p* = 0.033). Blood glucose (*p* = 0.01) and leucine levels (*p* = 0.001) were significantly lowered under MCT compared to baseline. For both oils, the serum concentration of acetate was higher (MCT, *p* = 0.0232; control, *p* = 0.0044), but lower for albumin (MCT, *p* = 0.0059; control, *p* = 0.0006) and stearic acid (MCT, *p* = 0.004; control, *p* = 0.0061) compared to baseline. HDL particle size decreased significantly under both oils (MCT, *p* = 0.002; control, *p* = 0.002).

In post-prandial state, two metabolites and four ratios were altered with statistical significance between Control DS and MCT DS phase (Supplementary Table 3). As in the pre-prandial state, Ala/BCAA ratio was decreased in the MCT DS phase. In proportion to the total fatty acids amount (%), the concentration of AA (*p* = 0.0357) was elevated, while oleic acid (*p* = 0.0182) and PUFA (*p* = 0.0184) were significantly lowered. MCT intake led to a thirty percent higher BHB serum concentration than Control-DS (*p* = 0.008). LDL particle size was significantly larger under MCT than to Control-DS (*p* = 0.0391). Under both oils, the triglyceride content of large HDL particles' triacylglyceride content was higher compared to baseline (MCT, *p* = 0.046; control, *p* = 0.047).

#### MCT response rate

Significant differences were found in the metabolic signature between MCT R (*N* = 5) to NR (*N* = 23). Four out of 123 measurands were significantly altered (Figure 3B, Supplementary Table 4). MCT-R showed higher relative (%) concentrations compared to the total fatty acid amount of AA (*p* = 0.0283) and overall Ω6 fatty acids (*p* = 0.0171), in their serum. Serum concentrations of pyruvate (MCT: 0.013 mmol/l vs. Control: 0.023 mmol/l; *p* = 0.0368) and TAG (MCT: 0.579 mmol/l vs. Control: 1.149 mmol/l; *p* = 0.0034) were significantly decreased in abundance, when compared to NR (Figure 3C). In 19 dogs, the dietary composition data could reconstructed (R = 4, NR = 15, Supplementary Table 5). On dry matter basis, dietary composition differed not significantly between MCT-R and MCT-NR (*p* = 0.579).

#### BHB-TAG ratio—The ketogenic BHB yield per fat

A ratio of BHB to TAG was calculated by dividing the serum concentration of BHB through TAG. The beneficial effects of MCFA on the brain are in humans mainly attributed to the ability of these fatty acids to provide energy sources in the form of ketone bodies, such as BHB. As BHB production depends on the overall fatty acid metabolism and not only MCFA, BHB/TGA ratio was calculated and presented.

**Figure 4:**
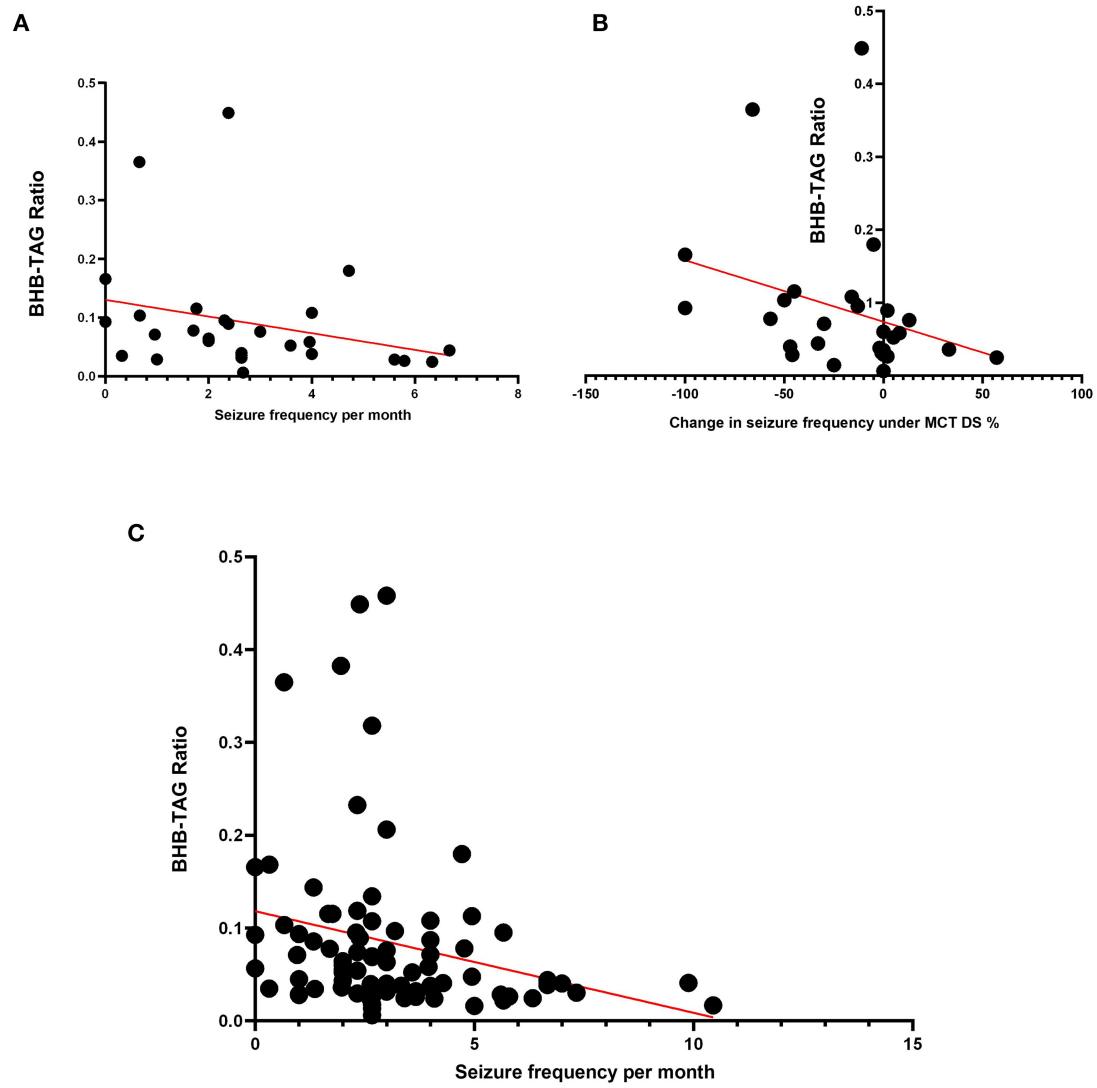
BHB-TAG Ratio: The BHB-TAG ratio was evaluated on its clinical relevance as a monitoring tool. **(A)** When MCT was consumed, a significant association between BHB-TAG ratio and seizure frequency per month (*N* = 28, *p* = 0.0150) was found. **(B)** The relative change in seizure frequency correlated negatively with BHB-TAG ratio (*N* = 28, r: −0.455, *p* = 0.015) under MCT intake. The lower the BHB-TAG ratio was, the higher the seizure frequency per month, or lower the change in seizure frequency under MCT. The higher the reduction in seizures (–%), the higher the BHB-TAG ratio. **(C)** Independent of diet or dietary supplementation, a significant negative correlation was found between the occurring seizure frequency per month and the BHB-TAG ratio (*N* = 84; r: −0.387; *p* = 0.0016).

The clinical relevance was then evaluated by correlating the documented seizure control and relative change after the dietary switch to MCT-DS (Supplementary Table 6). Significant association was found between BHB-TAG ratio and seizure frequency per months (*N* = 28, *p* = 0.0150) (Figure 4A). The relative change in seizure frequency under MCT consumption correlated negatively with BHB-TAG ratio (*N* = 28, r: −0.455, *p* = 0.015) (Figure 4B). The lower the BHB-TAG ratio was, the higher the seizure frequency per month, or lower the change in seizure frequency under MCT.

A dietary supplementation independent analysis (*N* = 84, MCT, Control, baseline) had shown again another significant negative correlation between the occurring seizure frequency per month and the BHB-TAG ratio (r: −0.387; *p* = 0.0016) (Figure 4C). Thus, the higher the BHB concentration is in relation to the TAG concentration, the better was the seizure control in our study population.

## Discussion

**Figure 5:**
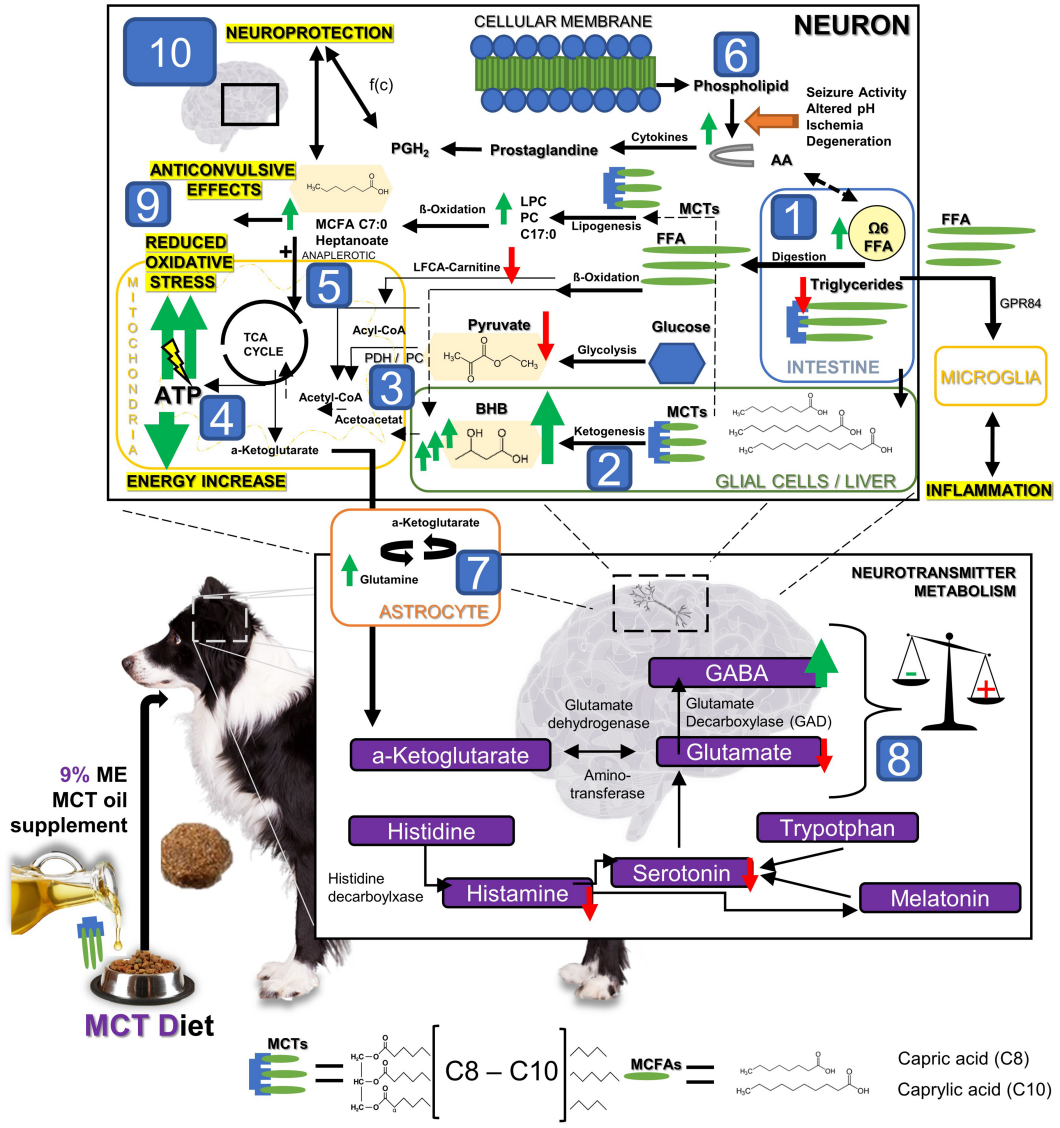
The hypothetical metabolic response to MCT oil supplementation: The dietary supplementation of MCT oil induces significant metabolic changes. Substantial changes in the lipid, energy and neurotransmitter metabolism have been detected in dogs with idiopathic epilepsy after 3 months under MCT DS as a nutritional, therapeutic approach. In brief, after oral intake, [1] MCT can be degraded in the intestinal lumen by lipases into MCFAs. MCFAs can be directly absorbed, transported into the liver and metabolized into C4 ketone bodies, such as beta-hydroxybutyrate (BHB). BHB levels [2] were significantly increased in all dogs during MCT consumption compared to the control oil phase. Based on these findings, MCT might lead to a significantly increased anaplerotic influx into the TCA cycle [3] and thereby evokes an additional production of adenosine triphosphatase (ATP) via the mitochondrial respiratory chain resulting in a compensation of epilepsy associated energy deficits [4]. Moreover, Pyruvate was found being especially decreased in MCT responders [3]. The compensation of energy deficits may thus also be given via (a) an increase in the enzymatic activity of the pyruvate dehydrogenase (PDH) activity, or/and (b) the promoted entry of oxaloacetate into the TCA by increased pyruvate carboxylase activity (PC). PDH and PC may be here both the key metabolic enzymes relevant for overcoming energy shortage under MCT consumption via the use of pyruvate. Furthermore, an additional *de-novo* lipogenesis from MCT into other MCFA via ß-oxidation leading to anaplerotic and neuroprotective effects has been hypothesized in the past [5]. Some prostaglandins deriving from arachidonic acid (AA) play significant roles in the processes of neurodegeneration and neuroinflammation, but also neuroprotection and regeneration. Prostaglandine can induce both depending on its concentration ratios of different types of prostaglandin [6]. The energy and neurotransmitter metabolism in neurons and astrocytes is tightly coupled [7]. Astrocytes take up GABA and glutamate from the synapse, and in turn, provide neurons with glutamine, an essential substrate for the re-synthesis of glutamate and GABA in the neurons. Based on our findings and previous research findings, we speculated that astrocytes are the main cellular compartment of MCFA metabolism and result in promoted astrocyte glutamine synthesis. The promoted glutamine supply from astrocyte metabolism of C8 and C10 MCFAs may lead then to elevated neuronal GABA synthesis [8], and thus aid in maintaining the inhibitory tone of the brain and provide another anticonvulsant mechanism of MCT oil supplementation. In summary, anticonvulsive effects may thus be provoked by I. compensating the energy shortage [2–5], II. influence on GABA/ glutamate balance via astrocyte metabolism [7–8], III. Reduction of antioxidative stress [9] via ATP increase and neuroprotective effects on the brain by MCT influencing metabolic pathways [10].

The metabolic and peripheral neurotransmitter changes induced through dietary supplementation of MCT oil were characterised in urine and serum in a canine population with idiopathic epilepsy (Figure 5). Although over 60% of the dogs experienced a reduction in seizure frequency, only 30% of the dogs had a reduction of 50% or more in seizure frequency (= MCT-R). The major finding of the current study was, that these responders had a different metabolic response to MCT oil than those dogs who showed no or less improvement. In summary, MCT consumption influenced significantly the amino-acid metabolism (serum alanine/BCAA and alanine/valine decreased compared to the control oil) and fat metabolism. While the serum amount of total fatty acids appeared similar during supplementation with the MCT-DS and control oil, the relative amounts of individual fatty acids differed. MCT consumption led to an increase in the relative concentrations of PUFA and AA compared to the control oil. BHB serum concentrations were significantly higher under MCT-DS than with the control oil and to baseline. The serum metabolic signature of MCT-R differed significantly to NR by higher relative concentrations of AA and overall Ω6 fatty acids as well as significantly decreased concentrations of pyruvate and TAG. Four out of nine neurotransmitters were altered considerably during the MCT phase: increased GABA concentration, accompanied by a significant GABAergic shift in the GABA/glutamate balance. Significantly lowered urinary concentrations of histamine, glutamate and serotonin were found during the MCT-DS phase in MCT-R.

In human medicine, urinary neurotransmitter excretion has been shown to effectively correlate not only with diverse neurological conditions (56–59), but also therapeutic effect of both dietary (60) and pharmacological interventions (61). Our urinary neurotransmitter analysis showed that both MCT-R and MCT-NR exhibited an increase in the GABA concentration and, an increase in the GABA/glutamate ratio compared to the control phase. Interestingly, a more significant reduction in glutamate was shown in MCT-R than in MCT-NR. GABA and glutamate are the primary inhibitory and excitatory neurotransmitters of the brain. The energy and neurotransmitter metabolism in neurons and astrocytes is tightly coupled, whereby astrocytes take up GABA and glutamate from the synapse, and in turn, provide neurons with glutamine, an essential substrate for the re-synthesis of glutamate and GABA in the neurons (62). Insufficient astrocyte glutamine synthesis is speculated to be closely linked to central nervous system (CNS) disease, with inhibition of glutamine synthesis resulting in seizures (63). Andersen and colleagues have recently demonstrated that astrocytes are the main cellular compartment of medium-chain fatty acid (MCFA) metabolism and result in promoted astrocyte glutamine synthesis (64, 65). The resultant increase in glutamine supply from astrocyte metabolism (66) of C8 and C10 MCFAs, may lead to elevated neuronal GABA synthesis, and thus aid in maintaining the inhibitory tone of the brain and provide an anticonvulsant mechanism of MCT oil supplementation. Given that GABA is formed through transamination of α-ketoglutarate to glutamate, which then undergoes decarboxylation by glutamic acid decarboxylase (GAD) to form GABA, our observed increase in GABA and decrease in glutamate could be therefrom consistent with previous studies (67). In addition, most recently dogs diagnosed with idiopathic epilepsy under anti-seizure pharmacotherapy were shown to have significantly higher GABA concentration in urine increased to a similar level as measured in healthy companions (68). However, even if plausible, this remains highly speculative, the measurements in urine do not let one conclude that the same changes can be found in the brain or reflect brain metabolism of MCFA being connected to neurotransmitter recycling in astrocytes. Based on our and previous *in-vitro* findings, we hypothesise that MCT may enhance anti-seizure drug-induced GABAgeric properties and thus leads to further seizure frequency reduction (65, 69–73). Future studies are necessary to directly measure metabolic processes within the brain tissue.

Our understanding of the pathophysiology of epilepsy is predominantly confined to inhibitory GABAergic and excitatory glutamatergic neurotransmission. However, a complex interplay exists between GABA, glutamate, histamine and serotonin. Monoamines such as histamine and serotonin are major modulators of the CNS and are thought to halt seizure activity (74). Elevated histamine levels suppress seizure activity and confer neuroprotection, whereas low levels are associated with seizures mediated through histamine H_1_ receptors (74). MCT administration resulted in decreased urine histamine levels in MCT-R compared to MCT-NR. This might be explained by the MCT-NR group having higher seizure frequency than MCT-R. In former studies, we have found a significant positive correlation between seizure frequency and histamine serum concentration (personal communication, 2022). Another explanation is that the MCT-NR and MCT-R differ in their degree of neuroinflammation. There is increasing evidence that supports a pathogenic role of neuroinflammation in various CNS diseases, including epilepsy (75). Histamine is known to modulate inflammatory processes critically and is a regulator of innate and adaptive immune responses. Previous studies have shown that histamine can stimulate microglia, the resident immune cells in the brain, resulting in activation and subsequent production of proinflammatory factors tumour necrosis factor (TNF)-alpha and interleukin-6 (IL-6) resulting in neuroinflammation (76). *In vitro*, decanoic acid at a very low concentration was able to activated the inflammatory receptor G protein-coupled receptor 84 (GPR84). As a receptor for MCFA with carbon length of C9 to C14, it is normally expressed at very low levels mainly in monocytes, neutrophils and microglia in the brain, but can be induced via lipopolysaccharide (77–79). Although knowledge about the role of proinflammatory receptors regarding anticonvulsant or other effects is still unclear, an altered neuroinflammation might be one explanation for some dogs with idiopathic epilepsy not responding to therapy (80). Therefrom, it might be possible that MCT can influence neuroinflammatory processes in some epileptic dogs via modulation of microglia activity in the brain. Although no adverse effects have been seen in the dogs under MCT consumption so far, more research about MCT effects on inflammation is needed.

Much like our observed changes in histamine, our results also showed a more significant reduction in urinary serotonin levels in MCT-R compared to MCT-NR. Serotonin, also known as 5-hydroxytryptamine (5-HT), is a monoaminergic neurotransmitter involved in fundamental brain functions such as the stress response, emotion and cognition. Accumulating evidence indicates that serotonergic neurotransmission modulates seizure susceptibility, and agents that generally elevate extracellular 5-HT levels, such as 5-hydroxytryptophan, inhibit both focal (limbic) and generalised seizures (81). At the same time depletion of brain 5-HT levels lowers the threshold for seizures (82). Our results are contradictory to what has been presented in the literature. However, there appears to be a disparity between the central (hippocampal) and peripheral (plasma) processes in regulating of 5-HT concentration. Maciejak et al. found that following intragastric administration of C8 and C10 fatty acids, there was an increased turnover of 5-HT in the hippocampus, detected through an increase in the concentration of 5-hydroxyindoleacetic acid (5-HIAA), the main metabolite of 5-HT, and 5-HIAA/5-HT ratios, but a decrease in plasma serotonin levels (83). This is thought to be due to the alteration of the tryptophan pathway activity, whereby the MCFA displace tryptophan from its binding sites on plasma albumins. This results in the free fraction of tryptophan being metabolised peripherally by hepatic tryptophan 2,3-dioxygenase while at the same time surplus tryptophan passes into the CNS. Given that tryptophan is the sole precursor of 5-HT, it is thought that once in the brain, tryptophan is converted into several different neurotransmitters, including 5-HT. It is therefore likely, that the observed changes of peripheral 5-HT in plasma are similar to the changes that we observed in urinary output. The fact, that epileptic dogs had significantly increased serotonin levels in urine (68), the hypothesis that MCT might have serotonergic modulating effects seems feasible. How central and peripheral serotonin levels in body are affected by dietary MCT, should be another focus in future clinical studies on the nutritional management of canine epilepsy.

The metabolic signature also differed significantly between MCT-R and MCT-NR. Supplementation of MCT-DS, when compared to the control oil, resulted in significant differences in four serum metabolites playing crucial roles in lipid and energy metabolism. In human medicine, available data suggest that brain energy metabolism is impaired in people with epilepsy (84–87). Accordingly, deficits in glucose metabolism of canine epileptic brains are also likely (37, 88, 89). Issues in brain energy supply might occur from (i) impaired glycolysis from plasma-derived glucose, (ii) decreased TCA activity and (iii) decreased production of amino-acid or lipid precursors (90).

Our results have shown that serum pyruvate concentrations were significantly decreased in MCT-R. Once pyruvate enters the mitochondria, it is typically integrated into the tricarboxylic acid (TCA) cycle via conversion through the enzyme pyruvate dehydrogenase (PDH) into acetyl-CoA. A shortage of glucose-derived intermediates and metabolites entering the TCA cycle has been found in several rodent epilepsy models (91, 92). Given that a reduced PDH activity was proposed being relevant in spontaneous seizure activity (92, 93), MCT might increase enzymatic activity (71). As capric acid has been proposed to be able to upregulate pyruvate dehydrogenase kinase 4 expression in skeletal muscle of mice (94), single MCFA in the DS might also be able to overcome the energy shortage in the brain permanently by genetic upregulation of the PDH expression and by surpassing the necessity of PDH by providing an alternative and direct energy source for TCA cycle (37). In brief, after oral intake, MCT can be degraded in the intestinal lumen by lipases into MCFAs. MCFAs can be directly absorbed, transported into the liver and metabolised into C4 ketone bodies, such as BHB. BHB levels were significantly increased in all dogs during MCT consumption compared to the control oil phase (40). Based on that, MCT leads to a significantly anaplerotic influx into the TCA cycle and thereby evokes an additional production of adenosine triphosphatase (ATP) via the mitochondrial respiratory chain resulting in a compensation of energy deficits (95). Another alternative pathway explaining a decrease in pyruvate may be the promoted entry of oxaloacetate into the TCA by increased pyruvate carboxylase activity (PC). In summary, both, PDH and PC, may be the key metabolic enzymes relevant for overcoming energy shortage under MCT consumption (37, 96).

Broken down to both MCFAs C8 and C10, previous *in-vitro* research showed an upregulated mitochondrial respiration (64), which could depend on the cell type, dose or serum concentrations (71, 97, 98). C8 was shown to increase the anaplerotic influx into the TCA in the brain of rats. The measured anaplerotic influx in the brain into the TCA cycle was found to be significantly associated with glutamine production (72), which would also support our previous hypothesis about the linkage to astrocytic metabolism ADDIN EN.CITE (72, 99). Most recent research showed as well, indication of active MCFA metabolism in astrocytes, while βHB is metabolised in a different cellular compartment (73).

Based on those findings, it is likely, that MCT may lead directly via ketogenesis and indirectly via anaplerotic influx to compensation for epilepsy-associated glucose hypometabolism. Future *in-vivo* and *in-vitro* research should focus on gaining further insight into MCT derived energy supply and its influence on the anaplerotic influx within the neuronal tissue.

When considering global shifts in lipid metabolism, due to MCT-diet consumption, the first findings were made in 2015 by Law and colleagues (49). Under an MCT containing kibble diet, dogs showed significantly increased abundances of C17:0 long fatty acids. In this study, we have shown that TAG concentrations are highly reduced in MCT-R serum. Triglycerides are the neutral storage form of diverse FA and occur primarily in hepatocytes. After eating, dietary lipids are hydrolyzed within the intestine. FA are re-esterified into TAG upon intestinal absorption, then packed into chylomicrons and delivered to the muscle and adipose tissue. The remaining TAG is then transported to the liver and processed into FA within the hepatocytes. In order to be metabolised, FAs are activated into acyl-CoA, which can then undergo oxidation or reconstituted into other lipids (100). Under normal conditions, the liver processes large quantities of FA and stores only small amounts as TAG. Upon fasting, TAG stored intracellularly are mobilised to release FA products, such as C4 ketone acetoacetic acid and BHB (101). Our results show changes in lipid metabolism, but how TAG metabolism is influenced long-term, over the year, by dietary added MCT requires future research.

In addition, the ratio of BHB to TAG was calculated, as tool to assess the ketogenic yield per fat depending on the established feeding regime (baseline without oil, with DS-1 or DS-2 oil). The clinical relevance was then investigated by analysing the association between the documented seizure frequency per months and the BHB-TAG ratio. To the authors knowledge, this has not been investigated in any species before. Independent from dietary supplementation (*N* = 84, MCT, Control, baseline) a significant negative correlation between the occurring seizure frequency per month and the BHB-TAG was found. This means, that the higher the BHB concentration was relative to the TAG concentration, the better was the seizure control in our study population. Exploring both DS separately, the same negative correlation was found under MCT, but not in the control oil feeding phase. As the baseline diet was documented to be stable over the entire study period, the fat profile was the only factor that was also altered, beside the type of oil. While the MCT oil contained C8 and C10, so MCFA, the used control oil was olive oil containing especially monounsaturated long chain fatty acids. Based on previous research, the type of fat may not play the only and significant role in the success of a ketogenic-orientated nutritional, therapeutic interventions (102–104), but also the fat profile and composition in relation to the patients complete diet (105–107). Future studies could consider therefrom BHB to TAG ratios and characterise the fat profile in the diet.

In this study, an increase in the relative concentrations of the Ω6 fatty acid AA and overall Ω6 fatty acids were detected in the serum of MCT-R. In humans, changes in AA metabolism have been extensively mapped in seizure states, wherefrom a correlation between AA concentrations and seizure control was often postulated (108). However, the investigations are not clear and quite conflicting. While some studies showed that KD-R (ketogenic diet responders) experienced a significant decrease in serum AA concentration compared to NR (109, 110), another has detected a 1.6- to 2.9-fold rise in AA levels in KD-R (111). The same controversy can also be discovered in rodent epilepsy models. While one study found that rats treated with KD experienced a decrease of serum AA concentration (112), another study in mice showed the same after oral Ω3 treatment, but with no change in seizure thresholds (113). From another perspective, AA can be enzymatically synthesised into diverse prostaglandins (PG) via membrane-associated cyclooxygenases (114). Some PGs play significant roles in the processes of neurodegeneration and neuroinflammation. In people with epilepsy, PGs are often found in higher concentrations in serum (115, 116). Depending on receptor subtype, cell population and receptor gene expression, PG can induce both neuroprotective and neuro-toxic effects in the brain (117). Therefrom, MCT could provide neuroprotection via influencing inflammatory stimuli from ongoing or past seizure activity. As a result, further elucidation is needed to evaluate how the MCT diet may be related to a serum AA increase and that could lead to improved seizure control.

Overall, the differences we have presented in serum metabolites and urinary neurotransmitters might be related to each other via the astrocyte metabolism. One critical function of astrocytes is the regulation of neurotransmitter homeostasis. Synaptically-released neurotransmitters, such as glutamate, GABA and glycine are taken up, metabolised and released as precursors back to the neurons (118). Moreover, MCFA may modulate astrocyte metabolism via activation of shuttle systems that provide fuel to neighbouring neurons in the form of lactate and ketone bodies (97, 119). Based on our finding and previous reported data from *in-vitro* research, we hypothesise that astrocytes may have a linking role between the neurotransmitter and energy homeostasis under MCT supplementation (72, 99). Further research is necessary, to explore this postulated linkage.

A few limitations of the present finding on metabolic and peripheral neurotransmitter changes induced through dietary supplementation of MCT oil should be considered. Due to the2 multicenter study design (52), variability and storage despite of standard operation procedures may impact our results. Furthermore, the number of dogs categorised as MCT-R was small (*N* = 5); thereby findings referring to this population must be interpreted cautiously. Another relevant limitation is the aspect, that the direct correlation between intracerebral and extracerebral metabolites (blood, urine) has been shown to a more or less extent in previous research. This has been discussed in detail in a recently published article from our group (68). Although the baseline diet was kept stable, urine collection was standardised and fasted serum samples were used to interrogate the global shifts in metabolism associated with diet consumption. Numerous factors can influence both body fluids.

Urinary neurotransmitter levels could arise from an additional synthesis outside the CNS. Besides the production in the PNS (120) or other organs, such the adrenal glands and kidneys (121), also the intestinal microbiome is capable of synthesising neuromodulating molecules (122–124). Although sex appears to influence urinary neurotransmitter profile in dogs (68), this should not be relevant in this study, as all samples have been evaluated in a paired analysis. Diverse factors might be able to affect metabolic profiling. The seasonal and diurnal variance of diverse metabolites have been identified in humans (125, 126). Although no clear evidence for that problem was found in dogs (127), it can be relevant for some metabolites. Another impact on our study results could come from the individual's metabolism (age, breed, sex, diet) (128) and in a subpopulation of epileptic dogs also the type of ASD medication (129). However, as the dietary supplement was the only changing factor in the study, and comparison was performed pairwise, significant impacts should not be present.

Metabolic perturbations in urine and serum reflect the response to MCT dietary supplementation and therefore help in understanding potential physiological mechanisms. In conclusion, this study provides further evidence about MCT-derived effects on the lipid and energy metabolism in dogs with IE. In addition, it highlights the first candidate biomarkers (BHB, AA%, Ω6 FA%, pyruvate, TAG; BHB/TAG ratio) being of potential use in a clinical setting for individual treatment monitoring and customised adjustments of nutritional therapeutic interventions. Overall, the results presented by this study provide further evidence of the value of MCT as dietary management for both human and canine drug-resistant epilepsy.

## Data availability statement

The raw data supporting the conclusions of this article will be made available by the authors, without undue reservation.

## Ethics statement

The animal study was reviewed and approved by the Royal Veterinary College Clinical Research Ethical Review Board (CRERB) (URN 2016 1558). Written informed consent was obtained from the owners for the participation of their animals in this study.

## Author contributions

BB planned the conduction of the study, carried out the main practical work, did the patients recruitment and the sample acquisition at each study centre, carried out statistical analysis, and interpreted the results. AW, AB-N, TJ, AK, and AT supported study conduction with recruitment and sample acquisition of dogs with idiopathic epilepsy at their study location. HV and RP designed the study. CO supported the sample acquisition and conducted the metabolomic analysis with physicochemical methods. HV reviewed and edited the manuscript. All authors contributed to the interpretation of the results, the manuscript revision, and approved the final manuscript.

## Funding

This study was funded by the AKC Canine Health Foundation to HV and RP (Grant 2252: Investigating a Ketogenic Medium-Chain Triglyceride (MCT) Supplement for the Treatment of Drug-Resistant Canine Idiopathic Epilepsy and Its Behavioral Comorbidities). BB received of a meritbased doctoral scholarship from the Hans-Böckler Foundation funded by the Federal Ministry of Education and Research – Germany (BMBF) and TL has been funded by BBSRC grant BB/P001874/1. NMR metabolomics analyses were funded by PetBiomics Ltd. This Open Access publication was funded by the Deutsche Forschungsgemeinschaft (DFG, German Research Foundation) – 491094227-Open Access Publication Funding and the University of Veterinary Medicine Hannover, Foundation.

## Conflict of interest

Author BB was CEO of BrainCheck.Pet^®^, a label and veterinary practise specialised into epilepsy and paroxysmal disorders in cats and dogs. Authors CO was an employee and HL the Chairman of Board of PetBiomics Ltd., a company providing NMR metabolomics testing for dogs. The remaining authors declare that the research was conducted in the absence of any commercial or financial relationships that could be construed as a potential conflict of interest.

## Publisher's note

All claims expressed in this article are solely those of the authors and do not necessarily represent those of their affiliated organizations, or those of the publisher, the editors and the reviewers. Any product that may be evaluated in this article, or claim that may be made by its manufacturer, is not guaranteed or endorsed by the publisher.

## Abbreviations

ASD: Anti-Seizure Drug
AA: Arachidonic Acid
BHB: Beta Hydroxybutyric Acid
C10: Decanoic Acid = Capric Acid
C8: Octanoic Acid = Caprylic Acid
DS: Dietary Supplement
FA: Fatty Acids
GABA: γ-Aminobutyric Acid
GPR: G-Protein Coupled Receptor
IE: Idiopathic Epilepsy
IVETF: International Veterinary Epilepsy Task Force
KBr: Potassium Bromide
Kcal: Kilocalories
KD: Ketogenic Diet
KD-R: Ketogenic Diet Responders
LEV: Levetiracetam
MCT: Medium Chain Triacylglyceride
ME: Metabolic Energy
NR: Non-Responders
PB: Phenobarbital
PC: Pyruvate Decarboxylase
PDH: Pryruvat Dehydrogenase
PG: Prostaglandin
PUFA: Polyunsaturated Fatty Acids
R, Responders, RVC: Royal Veterinary College
TCA: Tricarboxylic Acid
TAG: Triacylglyceride
QoL: Quality of Life.
