# Supplementary material for: Metabolic fingerprinting of dogs with idiopathic epilepsy receiving a ketogenic medium-chain triglyceride (MCT) oil
Source: Front Vet Sci. 2022 Oct 6;9:935430. doi: 10.3389/fvets.2022.935430 (PMC9584307; doi:10.3389/fvets.2022.935430)
Supplement: Supplementary file 1 [file Table_1.DOCX]

Supplementary Material

# Supplementary Tables

## Table 1:

Neurotransmitters (concentrations, ratios, change relative to baseline) shown to be significantly different in abundance between control and MCT DS phases, and between MCT responders versus non-responders. Urinary neurotransmitters were detected in Agilent 6460 liquid chromatography–mass spectrometry with Jet Stream electrospray ionization. The urine specimens were diluted according to their creatinine level. Significant differences were determined using match-paired Student’s t tests for parametric data and Wilcoxon matched-pairs signed rank test for non-parametric data.

| **Subgroup** | **Metabolite name** | **Molecular formula** | **URINARY CONCENTRATIONS** | | | **N** | **p-Value** |
| --- | --- | --- | --- | --- | --- | --- | --- |
|  |  |  | **Control-DS** | **MCT-DS** | **Unit** |  |  |
| **Control vs. MCT** | γ-Aminobutyric acid (GABA) | C_4_H_9_NO_2_ | 12.38 ± 7.48 | 15.52 ± 4.06 | µmol/g | 15 | 0.044 |

| **Subgroup** | **Metabolite name** | **Molecular formula** | **URINARY CONCENTRATIONS** | | | **N** | **p-Value** |
| --- | --- | --- | --- | --- | --- | --- | --- |
|  |  |  | **MCT-NR** | **MCT-R** | **Unit** |  |  |
| **MCT-NR vs. R** | Glutamate | C_5_H_9_NO_4_ | 56.1 ± 33.03 | 36 ± 6.28 | µg/g | 15 | 0.046 |
|  | Histamine | C_5_H_9_N_3_ | 110.2± 67.41 | 32.42 ± 20.5 | µg/g | 15 | 0.006 |
|  | Serotonin | C_10_H_12_N_2_O | 120.1± 33.4 | 71.26± 23.48 | µg/g | 15 | 0.012 |
|  |  |  |  |  |  |  |  |

| **Subgroup** | **Metabolite name** | **Molecular formula** | **CHANGE (%) RELATIVE TO BASELINE** | | | | | **N** | **p-Value** |
| --- | --- | --- | --- | --- | --- | --- | --- | --- | --- |
|  |  |  | **MCT-NR** | **%** | **MCT-R** | **%** | **Unit** |  |  |
| **MCT-NR vs. R** | Glutamate | C_5_H_9_NO_4_ | 4.1 ± 28.82 | ↑ | -19 ± 11.45 | ↓ | µmol/g | 15 | 0.046 |
|  | Histamine | C_5_H_9_N_3_ | 46.4 ± 69.64 | ↑ | - 49.98 ± 31.43 | ↓ | µg/g | 15 | 0.027 |
|  | Serotonin | C_10_H_12_N_2_O | 15.2 ± 22.42 | ↑ | -11.46 ± 9.91 | ↓ | µg/g | 15 | 0.026 |

| **Subgroup** | **Metabolite (M) ratio (M1:M2)** | **METABOLITE RATIO** | | | | **Relative change** | **N** | **p-Value** |
| --- | --- | --- | --- | --- | --- | --- | --- | --- |
|  |  | **Control-DS** | ***M1:M2*** | **MCT-DS** | ***M1:M2*** |  |  |  |
| **Control vs. MCT** | GABA: glutamate | 0.2 ±0.09 | 1 : 5 | 0.4 ±0.21 | 1 : 2.5 | 50% ↑ | 15 | 0.025 |

## Table 2

Pre-prandial serum metabolites (concentrations) shown to be significantly different in abundance between control and MCT DS phases (N= 28). Significant (S) metabolite concentrations were identified using match-paired Student’s t tests for parametric data and Wilcoxon matched-pairs signed rank test for non-parametric data. Data are shown either with median plus percentiles (25th, 75^th^) or mean plus standard deviation. The stars (*) mark groups being statistically compared and as found significantly different.

| **COMPARISON OF ALL METABOLITES BETWEEN BASELINE, CONTROL AND MCT** | | | | | | | | | | | | |
| --- | --- | --- | --- | --- | --- | --- | --- | --- | --- | --- | --- | --- |
| **Metabolite** | **Unit** | **Baseline** | | | **Control-DS** | | | **MCT-DS** | | | **p-Value** |  |
|  |  | **S** | **Median** | **Percentiles** | **S** | **Median** | **Percentiles** | **S** | **Median** | **Percentiles** |  |  |
| **Acetate** | mmol/l | * | 0.047 | [0.042 - 0.053] |  | 0.040 | [0.030 - 0.048] | * | 0.037 | [0.031 - 0.048] | 0.0232 |  |
|  |  | * |  |  | * |  |  |  |  |  | 0.0044 |  |
| **Ala/BCAA** | Ratio of alanine to total branched-chain amino acids |  | 0.659 | [0.53 - 0.84] | * | 0.712 | [0.57 - 0.91] | * | 0.601 | [0.49 - 0.83] | 0.0076 |  |
| **Ala/Val** | Ratio of alanine to valine |  | 1.352 | [1.03 - 1.71] | * | 1.428 | [1.19 - 1.88] | * | 1.171 | [1.04 - 1.72] | 0.0231 |  |
| **Albumin** | g/l | * | 26.050 | [24.44 - 28.10] |  | 25.180 | [23.87 - 26.96] | * | 24.330 | [23.21 - 26.41] | 0.0059 |  |
|  |  | * |  |  | * |  |  |  |  |  | 0.0006 |  |
| **AA** | mmol/l; Arachidonic acid, C20:4n-6 |  | 2.115 | [1.72 - 2.75] | * | 1.980 | [1.63 - 2.60] | * | 2.135 | [1.75 -2.95] | 0.0464 |  |
| **AA%** | Ratio of arachidonic acid to total fatty acids |  | 0.140 | [0.12 - 0.17] | * | 0.140 | [0.12 - 0.16] | * | 0.150 | [0.12 - 0.17] | 0.0081 |  |
| **BHB** | mmol/l;  β-Hydroxybutyric acid |  | 0.05 | [0.04 – 0.08] | * | 0.04 | [0.03 – 0.07] | * | 0.06 | [0.04 – 0.08] | 0.0410 |  |
|  |  |  | 0.07 ± 0.05 | | * | 0.06 ± 0.06 | | * | 0.07 ± 0.05 | | 0.0333 |  |
| **Glucose** | mmol/l | * | 4.690 | [4.04 - 5.23] |  | 4.450 | [4.06 - 4.91] | * | 4.494 | [3.93 - 4.90] | 0.0232 |  |
| **HDL-Diameter** | HDL particle size in nm | * | 10.620 | [10.44 - 11.27] |  | 10.490 | [10.36 - 10.89] | * | 10.510 | [10.33 - 11.02] | 0.0002 |  |
|  |  | * |  |  | * |  |  |  |  |  | 0.0020 |  |
| **Leucine** | mmol/l | * | 0.117 | [0.10 - 0.14] |  | 0.119 | [0.09 - 0.16] | * | 0.129 | [0.12 - 0.15] | 0.0014 |  |
| **PUFA%** | Ratio of polyunsaturated fatty acids to total fatty acids |  | 0.499 | [0.48 - 0.52] | * | 0.500 | [0.47 - 0.52] | * | 0.550 | [0.49 - 0.53] | 0.0162 |  |
| **SFA%** | Ratio of saturated fatty acids to total fatty acids |  | 0.373 | [0.36 - 0.38] | * | 0.373 | [0.36 - 0.38] | * | 0.371 | [0.35 - 0.37] | 0.0450 |  |
|  |  | * |  |  |  |  |  | * |  |  | 0.0162 |  |
| **SteA%** | Ratio of stearic acid (C18) to total fatty acids | * | 0.181 | [0.18 - 0.19] |  | 0.178 | [0.17 - 0.18] | * | 0.177 | [0.17 - 0.18] | 0.0021 |  |
|  |  | * |  |  | * |  |  |  |  |  | 0.0111 |  |

## Table 3

Post-prandial serum metabolites (concentrations) shown to be significantly different in abundance between control and MCT DS phases (N=10). Significant (S) metabolite concentrations were identified using match-paired Student’s t tests for parametric data and Wilcoxon matched-pairs signed rank test for non-parametric data. Data are shown either with median plus percentiles (25th, 75^th^) or mean plus standard deviation. The stars (*) mark groups being statistically compared and as found significantly different.

| **COMPARISON OF ALL METABOLITES BETWEEN BASELINE, CONTROL AND MCT** | | | | | | | | | | | | |
| --- | --- | --- | --- | --- | --- | --- | --- | --- | --- | --- | --- | --- |
| **Metabolite** | **Unit** | **Baseline** | | | **Control-DS** | | | **MCT-DS** | | | **p-Value** |  |
|  |  | **S** | **Median** | **Percentiles** | **S** | **Median** | **Percentiles** | **S** | **Median** | **Percentiles** |  |  |
| **Ala/BCAA** | Ratio of alanine to total branched-chain amino acids |  | 0.660 | [0.55 - 0.74] | * | 0.753 | [0.60 - 0.78] | * | 0.605 | [0.56 - 0.70] | 0.0184 |  |
| **AA%** | Ratio of arachidonic acid to total fatty acids |  | 0.155 | [0.13 - 0.18] | * | 0.139 | [0.12 - 0.18] | * | 0.178 | [0.14-0.19] | 0.0357 |  |
| **BHB** | mmol/l;  β-Hydroxybutyric acid |  | 0.04 ± 0.01 | | * | 0.04 ± 0.01 | | * | 0.06 ± 0.03 | | 0.0088 |  |
| **LDL Diameter** | LDL particle sizes in nm |  | 22.52 | [22.44 - 22.66] | * | 22.59 | [22.28 - 22.73] | * | 22.63 | [22.46-22.95] | 0.0391 |  |
| **L-HDL-TAG** | Triglyceride content of Large HDL particles in mmol/l | * | 0.017 | [0.02 -0.03] | * | 0.024 | [0.02 - 0.03] |  | 0.024 | [0.02 - 0.04] | 0.046 |  |
|  |  | * |  |  |  |  |  | * |  |  | 0.047 |  |
| **OleA%** | Ratio of oleic acid to total fatty acids |  | 0.131 | [0.11-0.15] | * | 0.141 | [0.13 - 0.17] | * | 0.128 | [0.11 - 0.16] | 0.0182 |  |
|  |  | * |  |  | * |  |  |  |  |  | 0.0096 |  |
| **PUFA%** | Ratio of polyunsaturated fatty acids to total fatty acids |  | 0.496 | [0.47-0.52] | * | 0.474 | [0.45 - 0.49] | * | 0.491 | [0.47-0.51] | 0.0184 |  |
|  |  | * |  |  | * |  |  |  |  |  | 0.0095 |  |

## Table 4

Pre-prandial serum metabolites (concentrations) shown to be significantly different in abundance between MCT responders (R, N=5) and non-responders (NR, N=10). Analysis was conducted using a validated, canine-specific proton nuclear magnetic resonance spectroscopy-based metabolomics platform quantifying 123 measurands. Significant (S) metabolite concentrations were identified using Mann-Whitney-U-Test for independent data sets. Data are shown with median plus percentiles (25th, 75^th^). The stars (*) mark groups being statistically compared and as found significantly different.

| **COMPARISON OF ALL METABOLITES BETWEEN BASELINE, CONTROL AND MCT** | | | | | | | | | | | | |
| --- | --- | --- | --- | --- | --- | --- | --- | --- | --- | --- | --- | --- |
| **Metabolite** | **Unit** | **Baseline** | | | **MCT-R** | | | **MCT-NR** | | | **p-Value** |  |
|  |  | **S** | **Median** | **Percentiles** | **S** | **Median** | **Percentiles** | **S** | **Median** | **Percentiles** |  |  |
| **AA%** | Ratio of arachidonic acid to total fatty acids |  | 0.155 | [0.13 - 0.18] | * | 0.166 | [0.15 - 0.18] | * | 0.140 | [0.12 - 0.16] | 0.0283 |  |
| **Omega6%** | Ratio of omega-6 fatty acids to total fatty acids |  | 0.443 | [0.43-0.46] | * | 0.470 | [0.46 - 0.48] | * | 0.436 | [0.42 - 0.46] | 0.0171 |  |
| **Pyruvate** | mmol/l |  | 0.021 | [0.016 - 0.024] | * | 0.013 | [0.012 - 0.020] | * | 0.023 | [0.017-0.027] | 0.0386 |  |
| **TAG** | mmol/l |  | 0.962 | [0.72 - 1.15] | * | 0.579 | [0.35 - 0.82] | * | 1.149 | [0.83 - 1.56] | 0.0034 |  |

## Table 5

The average macronutrient profile of the base diet from 19 dogs of the trial population calculated from analytical data provided by each company. The profile between MCT Responders and non-responders was not significantly different (p=0.579).

|  | % in 100 g/ 1000 g = 1 kg | | | | | | |  |
| --- | --- | --- | --- | --- | --- | --- | --- | --- |
| **DOG** | **Crude Protein** | **Crude Fat** | **Crude Fibers** | **Crude Ash** | **Moisture** | **Calcium** | **Phosphorus** |  |
| **3** | 21.00% | 12.00% | 2.50% | 6.00% | 8.50% | 1.04% | 0.73% |  |
| **5** | 16.00% | 10.25% | 2.00% | 4.50% | 43.25% | 1.40% | 0.72% |  |
| **6** | 26.00% | 11.00% | 16.00% | 8.00% | 10.00% | 0.80% | 0.60% |  |
| **8** | 18.50% | 8.00% | 2.50% | 7.50% | 8.00% | 2.06% | 1.05% |  |
| **9** | 30.00% | 11.00% | 6.80% | 5.10% | 8.00% | 0.82% | 0.64% |  |
| **10** | 25.00% | 14.00% | 8.00% | 3.00% | 8.00% | 1.60% | 1.00% |  |
| **12** | 26.20% | 16.00% | 2.60% | 5.50% | 8.00% | 0.95% | 0.75% |  |
| **15** | 22.70% | 12.80% | 1.30% | 6.20% | 10.00% | 0.99% | 0.72% |  |
| **16** | 18.50% | 7.50% | 2.50% | 7.00% | 8.00% | 1.30% | 0.87% |  |
| **18** | 17.39% | 15.76% | 1.30% | 5.50% | 10.00% | 0.95% | 0.66% |  |
| **19** | 20.00% | 7.50% | 3.00% | 5.00% | 10.00% | N/A | N/A |  |
| **20** | 18.00% | 7.50% | 3.50% | 7.00% | 10.00% | 0.90% | 0.60% |  |
| **24** | 20.00% | 8.00% | 3.50% | 7.50% | 10.00% | 1.20% | 0.80% |  |
| **27** | 26.00% | 16.00% | 2.00% | 7.90% | 10.00% | 1.50% | 1.00% |  |
| **28** | 22.00% | 12.00% | 3.00% | 7.00% | 10.00% | 1.50% | 1.00% |  |
| **30** | 16.50% | 8.75% | 1.70% | 4.45% | 42.00% | 0.75% | 0.50% |  |
| **32** | 26.00% | 16.00% | 9.50% | 3.50% | 8.00% | 2.30% | 1.50% |  |
| **33** | 23.00% | 16.50% | 1.20% | 6.50% | 10.00% | 0.30% | 0.25% |  |
| **34** | 22.70% | 12.80% | 1.30% | 6.20% | 10.00% | 0.99% | 0.72% |  |
| **TOTAL:** | **21.87%** | **11.76%** | **3.91%** | **5.97%** | **12.72%** | **1.19%** | **0.78%** |  |
| **MCT NR:** | **22.01%** | **12.03%** | **4.16%** | **6.20%** | **9.23%** | **1.00%** | **0.82%** | **P = 0.579** |
| **MCT R:** | **21.30%** | **10.70%** | **2.95%** | **5.06%** | **25.81%** | **1.02%** | **0.78%** |  |

## Table 6

Evaluation of the BHB-TAG Ratio

|  | | | **EVALUATION OF BHB-TAG RATIO** | | | | | | |
| --- | --- | --- | --- | --- | --- | --- | --- | --- | --- |
| **Variable 1** | **Variable 2** | | **Phase** | **N** | **Spearman** | | **p-Value** | **Significance** |  |
|  |  |  |  |  | **r** | **Interval** |  |  |  |
| **BHB – TAG Ratio** | Seizure frequency per months | | MCT | 28 | -0.3749 | -0,6627 to 0,009519 | 0.0240 | * |  |
|  |  |  | Control | 28 | -0.2786 | -0,5978 to 0,1169 | 0.151 | - |  |
|  |  |  | Baseline | 28 | -0.4255 | -0,6952 to -0,05074 | 0.0494 | * |  |
|  |  |  | MCT, C, B | 84 | -0,387 | -0,5658 to -0,1735 | 0.0016 | ** |  |
|  | % change in seizure frequency  related to the DS | | MCT | 28 | -0.4550 | - 0.7137 to -0.08724 | 0.0150 | * |  |
|  |  |  | Control | 28 | 0.466 | 0.1018 to 0.7208 | 0.0123 | * |  |
